# The influence of accent on the evaluation of trust-building efforts during conflict

**DOI:** 10.1371/journal.pone.0311373

**Published:** 2024-11-13

**Authors:** Leigh H. Grant, Alexandra Shahwan, Ifat Maoz, Boaz Keysar

**Affiliations:** 1 Department of Psychology, University of Chicago, Chicago, Illinois, United States of America; 2 The Program for Gender and Diversity Studies, Hebrew University of Jerusalem, Jerusalem, Israel; 3 Department of Communication and Journalism, Hebrew University, Jerusalem, Israel; University of Missouri Columbia, UNITED STATES OF AMERICA

## Abstract

The Israeli-Palestinian conflict has been an ongoing source of violence in the Middle East, claiming the lives of tens of thousands of people. As of late violence has escalated, with this year being one of the deadliest years in the conflict in decades. Therefore, now more than ever finding ways to bridge divides is essential to reduce the human suffering associated with the conflict. In this study we evaluated the impact of an important element of communication: accent. We demonstrate that the accent through which trust-building initiatives are communicated can inadvertently sway public opinion regarding their benefits. Jewish-Israelis listened to the same trust-building proposal communicated by a Palestinian delegate with varying degrees of Palestinian Arabic-accented Hebrew. When the same proposal came from a Palestinian delegate with a heavier accent, Jewish-Israelis thought this proposal was significantly worse for Israel than when it was offered by a Palestinian delegate who spoke Hebrew with no detectable, non-native accent. This effect was explained by differences in how the Palestinian delegate was judged depending on his accent. When the delegate spoke with heavier, Arabic-accented Hebrew, he was judged more harshly than when he spoke Hebrew with no such accent, which in turn reduced how favorably Jewish-Israelis evaluated the proposed measures. Our findings show that the way in which trust-building measures are communicated can shape how they are received and thus has direct implications for diplomatic efforts.

## Introduction

Violent conflict is a pernicious issue that continues to plague the modern world. It is estimated that over 2 billion people live in conflict-affected areas, among whom 274 million need humanitarian aid and 84 million have been displaced [1]. Among these conflicts, the ongoing Israeli-Palestinian conflict has been one of the longest continuously ongoing conflicts. Over the course of the last century, this conflict has claimed the lives of tens of thousands of people while displacing millions, with 2023/24 being on track to be one of the deadliest periods in decades [2]. Therefore, now more than ever finding ways to de-escalate the ongoing violence and to work towards peace are of upmost importance as a means of reducing the human suffering associated with a protracted conflict. In this study, we aim to investigate the extent to which different communicative practices may help or hinder efforts to deescalate conflict.

While numerous sociopolitical factors have fueled the continuation of the Israeli-Palestinian conflict [3], one rarely discussed issue which is common in intergroup conflicts is the lack of a shared native tongue through which to communicate. This is a significant issue, as parties who lack a shared native language must first overcome this communication barrier before they can begin working towards peace. One commonly used method is to communicate in the native language of the other side, which may prove beneficial as it signals to the recipient that the speaker is investing more effort in order to bridge cultural barriers to boost communicative effectiveness [4]. However, choosing to speak in a language that is native to the recipient but not to the speaker often entails communicating through a non-native accent. In this study we examine the consequences of delivering a trust-building proposal when speaking with varying degrees of non-native accented speech in the context of the Israeli-Palestinian conflict.

### Theoretical accounts

There are reasons that communicating trust-building measures through a relatively heavy, non-native accent may affect how favorably trust-building measures are received. We consider two accounts: I. Accent bias account and II. Language incongruency account.

#### Accent bias account

This account is based on extensive research which has demonstrated that individuals make more negative social evaluations of non-standard speakers than those who communicate using a more standard speech style [5]. Specifically, those who have non-standard speech–such as non-native or regionally marked accents–are typically evaluated more negatively across a variety of dimensions, including traits associated with status, solidarity, and dynamism [6]. Therefore, it may be the case that providing trust-building measures through speech without a detectable non-native accent will be more favorably received. This account, then, predicts that people would more harshly judge speakers when they have a heavy non-native accent, which in turn will reduce the perceived value of the proposal for one’s own side.

Several theories have been offered to explain this tendency to evaluate speakers who have non-standard accents more negatively. First, non-standard accents are more disfluent. There is evidence that the relative disfluency of non-standard speech elicits a more negative emotional response which in turn negatively impacts social evaluations of the speaker [7]. Second, the extent to which a speaker has a detectable, non-standard accent can represent a greater divergence in speech style from the listener who has a more standard accent, with greater speech divergence generally resulting in more negative social evaluations [8]. Lastly, people with non-standard accents are often from underrepresented or historically disadvantaged communities, of which people sometimes hold negative, stereotypical views. Therefore, individuals who speak with a heavier, non-standard accent typically associated with that group can be viewed as a more prototypical group member [9] which in turn can lead to more negative, stereotypical evaluations of the speaker [10].

Therefore, if people form a more negative impression of a speaker who communicates through a non-native accent, this may result in the proposal itself being less favorably received than when it is offered by a speaker without a detectable non-native accent. Importantly, though, because there are several proposed mechanisms for why this accent bias might occur, there are a few different causal pathways that this account predicts. If negative social evaluations are triggered by processing disfluency, this would predict that differences in processing fluency would lead to differences in how positively the listener feels after listening to the proposal. This, in turn, would impact the evaluations of the proposal. Alternatively, if listeners view the heavier accented speaker as a more prototypical member of his group, leading to increased negative stereotyping, then this would predict that differences in perceived group prototypicality would lead to differences in social evaluations which in turn would impact proposal evaluations. These paths of the accent bias account are not mutually exclusive and both predict that the proposal will be more negatively evaluated when offered by a heavier, non-native accented speaker.

#### Language incongruency account

This account assumes that evaluation of the speaker depends on the perceived level of congruency between the accent and the identity of the speaker. This builds on evidence that language incongruency triggers more negative evaluations of speakers. For instance, Ross, Shortreed, and City (1990) found that Japanese listeners perceived foreigners with the most native-like Japanese as less cooperative, polite, and empathetic than foreigners using less native-like speech such as codeswitching or foreigner talk [11]. They theorized that foreigners using more native-like Japanese violated expectations for how someone of their background should communicate which resulted in more negative speaker evaluations. This is echoed by Preston (1981), who highlights that when a speaker communicates in an unexpected way–such as when a known non-native speaker has a native-like speech style–listeners can view this as being performative or worse, manipulative, which in turn can lead to harsher evaluations of the speaker than when he has a more congruent, non-native way of speaking [12].

Hence, if a member of the opposing side in conflict who does not share your native tongue offers a proposal using native-like accented speech, the incongruency between the language and known identity of the speaker may backfire. Specifically, the incongruency may violate listener expectations of how the speaker should communicate, which may result in the speaker being more negatively evaluated. This, in turn, may result in the proposal being viewed less favorably by the receiving party than when the speaker communicates using more congruent, non-native accented speech.

### Current study

To test the predictions of the two accounts, we presented Jewish-Israeli respondents with a trust-building proposal from a Palestinian delegate who spoke either with a heavily Arabic-accented Hebrew (Heavy), mildly Arabic-accented Hebrew (Mild), or Hebrew with no detectable non-native accent (Native-like). We used the matched-guise technique [13], in which participants in the different accent conditions listened to the same proposal, spoken with varying accent characteristics, and measured their evaluations of the proposal. We included both mild and heavy Arabic-accented Hebrew condition, as research on the influence of accent on social evaluations has found that heavier, non-native accented speakers are considered more prototypical of their group and elicit more negative reactions than their more mildly accented counterparts [14].

This study was approved by the University of Chicago Institutional Review Board (IRB23-0312), and participants digitally agreed to a written consent form prior to participating. This study was preregistered on Open Science Framework (https://osf.io/yz9p5) and the full data and analysis script will be available upon publication.

## Materials and methods

### Participants

450 Jewish-Israeli native Hebrew speakers participated in an online survey through the survey panel Midgam (https://www.midgampanel.com/) between May 31^st^ and June 2^nd^, 2023. All participants were prescreened to ensure they were native Hebrew speakers and were 18 years or older and digitally agreed to a written consent form prior to participating. One person was excluded from analysis due to evidence of random responding, identified based on providing inconsistent responses across several study items resulting in outlier responses to the index measures. This left a final sample of 449 participants (see Table 1 for demographic information).

**Table 1:** Demographic characteristics of participants.

Age	Gender	Education	Religious Identification	Political Attitudes
44.23 (13.47)	44.10% Women	52.78% Undergraduate degree or higher	56.79% Secular	42.31% Right
			22.49% Traditional	36.30% Centre
			15.15% Religious	21.38% Left
			5.57% Ultra-Orthodox	

Participants were randomly assigned to listen to a trust building proposal from a Palestinian delegate in one of three conditions: Hebrew with no Arabic accent (Native-like; *n* = 147), mildly Arabic-accented Hebrew (Mild; *n* = 145), or heavily Arabic-accented Hebrew (Heavy; *n* = 157). Within each accent condition, participants were randomly assigned to listen to one of two different speakers who were previously pretested to ensure they are perceived as having a similar degree of Arabic-accented Hebrew (see S1 File and S1 Table for more details on the speaker selection process).

### Materials

**Fig 1:**
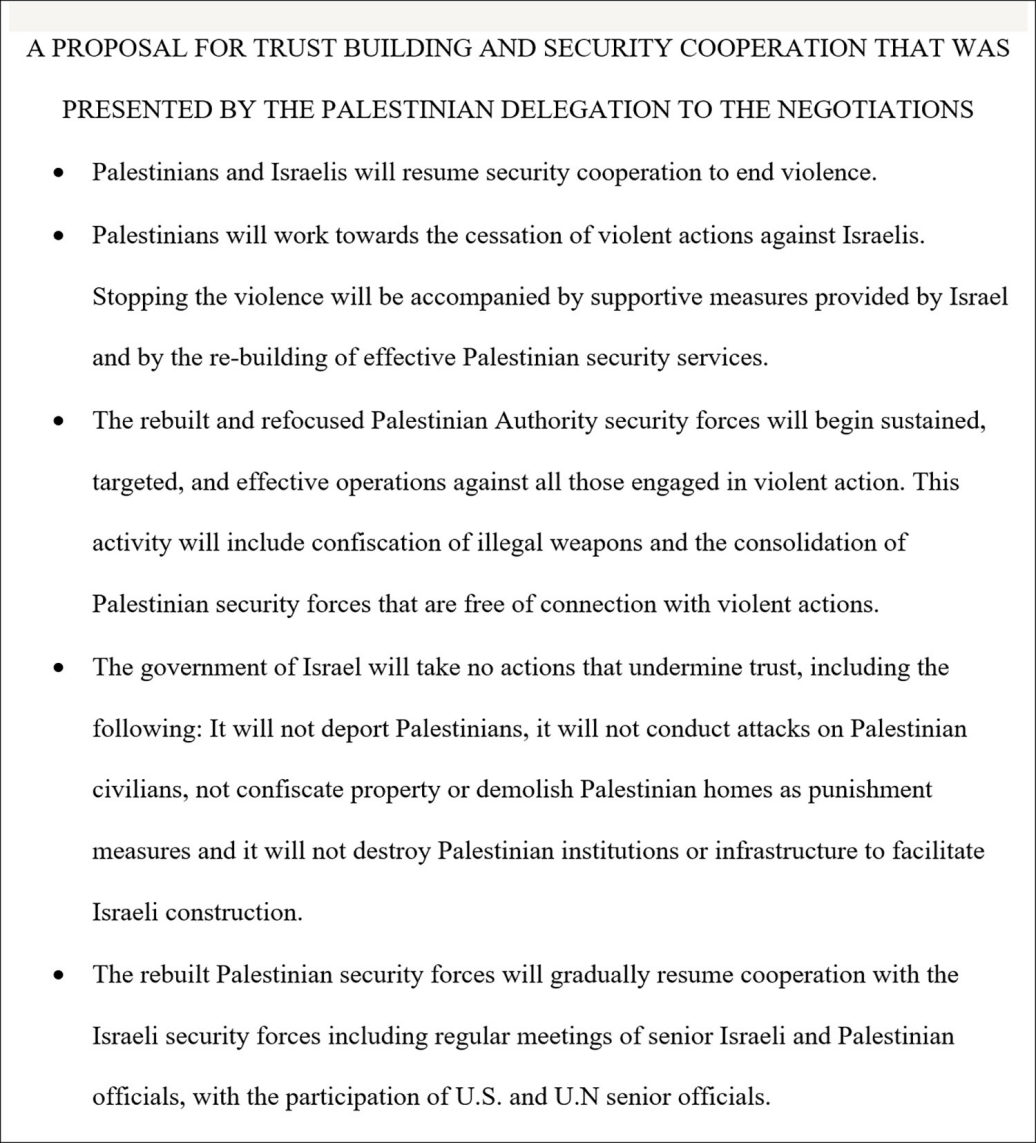
Trust-building proposal presented to Jewish-Israelis by the Palestinian delegation.

An Israeli-Palestinian trust-building proposal was developed focusing on five major issues relevant to the ongoing Israeli-Palestinian conflict, adapted from the proposal first generated by Grant, Maoz, and Keysar (2022) [15]. The overarching goal of the proposal was to build trust between Israelis and Palestinians, through ending any overt acts of violence against each other as well as working together to rebuild a coordinated security effort. All research materials–including instructions, questionnaires, and the consent form—was initially written in English and then translated to Hebrew using a professional translation service. The materials were then reviewed for fluency by a separate Hebrew-English bilingual before final corrections were made by two of the authors who are fluent Hebrew-English bilinguals. The final Hebrew text of the materials were used for all participants in the study. See Fig 1 for the trust-building proposal in English and Open Science Framework (https://osf.io/t58ac/) for the trust-building proposal in Hebrew.

### Procedure

Participants were first prescreened to ensure they were native Hebrew speaking, Jewish-Israelis that were born and raised in Israel and had normal or corrected to normal hearing. Participants who passed these requirements were then automatically redirected into the main study, and randomly assigned to listen to the proposal from a Palestinian delegate in one of the three accent conditions (Heavy, Mild, or Native-like). Within each accent condition participants were randomly assigned to listen to one of the two speakers who were rated as having similar perceived accent in the norming study (see S1 File and S1 Table for more details on the speaker selection process).

To make sure their audio equipment was functioning, participants first completed a brief audio check. They received a brief recording instructing them to select a color name from a list of options. Those who did not pass the audio check after two attempts were unable to advance to the main study. Those who passed the audio check were told they would be listening to the main points of a trust-building and security cooperation proposal that was offered by a representative from a Palestinian delegation. They were instructed to listen carefully as they would be asked questions about the offer later in the study. They then heard the proposal. Participants were able to listen to it only once and then advance only once the proposal ended.

Next, participants answered a series of questions assessing the extent to which they evaluated the proposal as favorable for each side, adapted from Grant, Maoz, and Keysar (2022). Specifically, to examine the extent to which participants perceived the proposal as favorable for Israel, they reported the extent to which they perceived the proposal as pro-Israeli, fair to Israelis, the extent to which they agreed with the proposal, and the extent to which they thought Israelis more broadly would agree with the proposal. To examine how they perceived the proposal as favorable for Palestinians they reported the extent to which they perceived the proposal as pro-Palestinian and as fair to Palestinians. Participants then evaluated the extent to which the proposal is a good basis for negotiations. Finally, then received a brief attention check that asked them to select the number ‘5’ from a scale presented below the question. Participants who failed this attention check were removed from the study. All measures were completed on a scale from 1 (*not at all*) to 7 (*to a very high extent*).

Following the evaluation section, participants completed two sections assessing how they felt after listening to the proposal and how they felt about the delegate. The order of these two sections was counterbalanced so that for half of participants, the delegate evaluation section came first whereas for the other half of participants the emotional response to the proposal section came first. To assess their emotional response to the proposal, participants reported the extent that the proposal made them feel different emotional states, including both negative emotions (anger, hatred, hostility, fear, concern, a sense of threat, disgust, contempt) and positive emotions (sympathy, empathy, hope, and optimism) [16]. To assess their evaluation of the delegate, participants rated the extent to which they viewed the Palestinian delegate as having different traits, including competence (intelligent, educated, competent, successful), trustworthiness (trustworthy, credible, sincere, and honest), and warmth (friendly, warm, and good-natured) as well as the extent to which they thought the speaker was coercive (manipulative, looking after his own interests, demanding) [6, 11, 12]. Participants also rated how close they think the Palestinian representative is to the Palestinian people as a measure of prototypicality of the speaker, using a 1 to 6 scale of increasingly overlapping circles to indicate the degree of closeness between the Palestinian representative and other Palestinians [17]. Except for the prototypicality measure, all scales ranged from 1 (*not at all*) to 7 (*to a very high extent*).

After completing the main study measures, participants were asked a series of questions evaluating the ease of understanding the Palestinian delegate as well as two comprehension questions regarding the proposal. The ease of understanding measure included two questions in which participants rated the extent to which the speaker was easy to understand and clear to understand on a scale of 1 (*not at all*) to 7 (*to a very high extent*) [7]. For the comprehension questions, participants were asked to identify the topic and source of the proposal. Lastly, participants responded to a series of demographic questions. These questions included two items on their religious background and political attitudes, as well as a measure assessing how frequently they interact with Palestinians in their everyday life. For exact wording of the study materials, refer to the materials on Open Science Framework upon publication (https://osf.io/t58ac/). The data was collected before the Hamas attack on Israel on October 7, 2023 and the subsequent war.

## Results

All analyses were conducted using a two-way ANOVA examining the main effect of Accent (Heavy | Mild | Native-like). Because we planned to analyze aggregated indexes as opposed to individual Likert items, we selected two-way ANOVAs over alternate models such as cumulative link mixed effects models in which the Likert data would be analyzed as ordinal data [18–20]. In instances in which a significant effect of Accent was detected further post-hoc Tukey contrasts were performed to determine which accent conditions significantly differed from one another.

Additionally, in our initial analyses we assessed two possible moderating factors in our models. First, because political attitudes influence how individuals respond to conflict-relevant information, we initially included the main effect and interaction effect of reported political attitudes on the extent to which the proposal was viewed as good for Israelis or Palestinians to determine whether preexisting political attitudes serve as a moderating factor. Because there was a significant main effect of political attitudes but no interaction with accent condition, the models reported below were simplified, controlling for political attitudes as a covariate. The results hold even when political attitudes are removed from the models. Second, we tested whether intergroup contact—how often Jewish-Israelis interact with Palestinians—moderated how individuals responded to the proposal, however neither the main effect nor interaction of frequency of intergroup contact were significant across any of the main dependent variables. Therefore, this factor was dropped from the models reported below.

### Proposal evaluation

**Fig 2:**
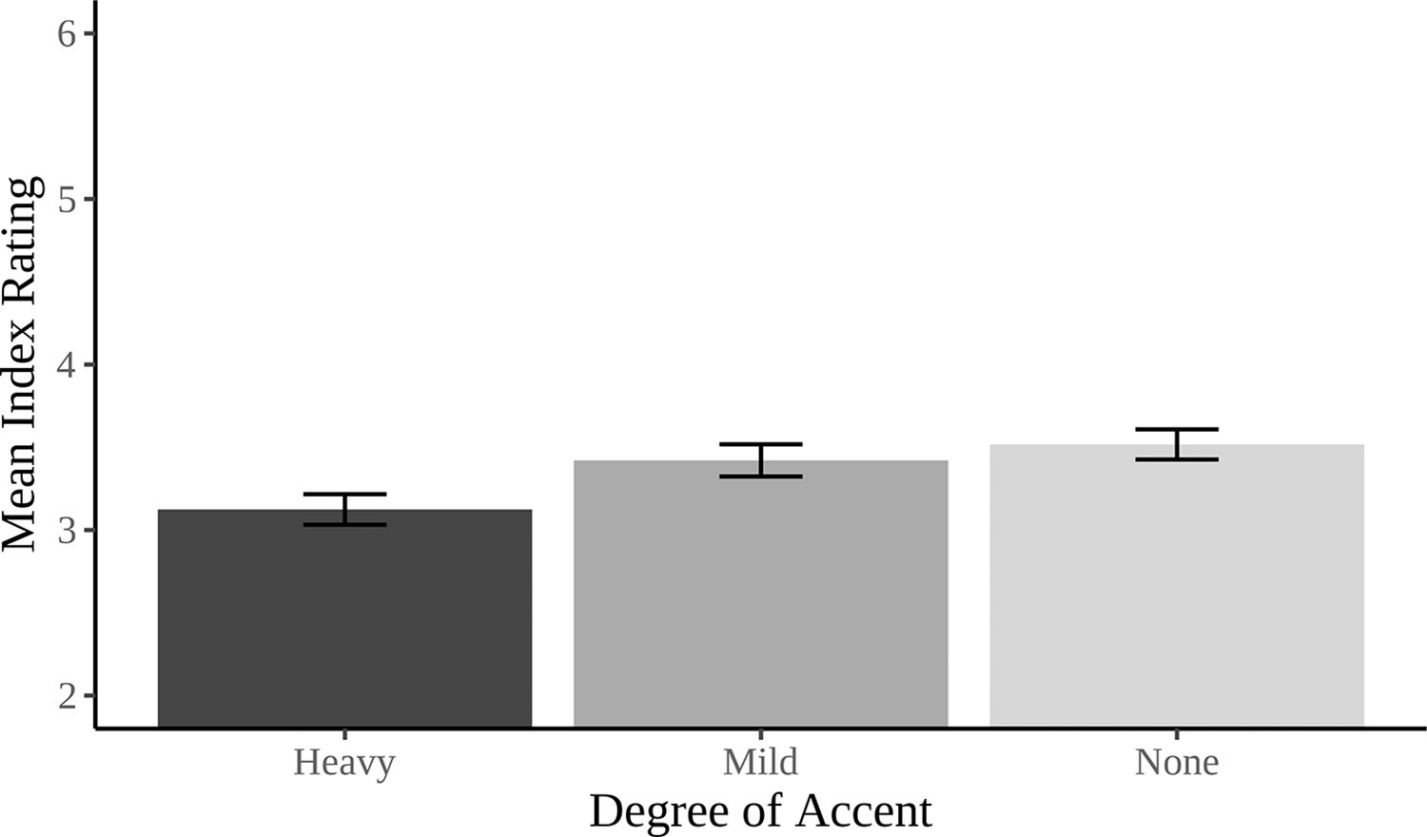
Mean index ratings of the extent to which the proposal was perceived as pro-Israeli as a function of the accent of the speaker presenting the proposal.

A pro-Israeli index was created by collapsing the pro-Israeli, fair to Israelis, self-agreement, and general Israeli agreement measures (Cronbach’s α = 0.89) to examine if accent influences the extent to which the proposal is viewed as beneficial for Israel. There was a main effect of Accent (*F*(2, 445) = 3.71, *p* = 0.03, η_p_^2^ = 0.01), with further post-hoc contrasts revealing a significant difference in proposal evaluations between the Native-like (*M* = 3.52, *SD* = 1.36) and Heavy (*M* = 3.12, *SD* = 1.38) accent conditions (*t* = 2.47, *p* = 0.04, *d* = 0.23). The Mild accent condition (*M* = 3.42, *SD =* 1.46), was not significantly different from the Native-like (*t* = 1.11, *p =* 0.51, *d =* 0.06) or the Heavy (*t* = 1.32, *p* = 0.38, *d* = 0.17) accent conditions (see Fig 2).

**Fig 3:**
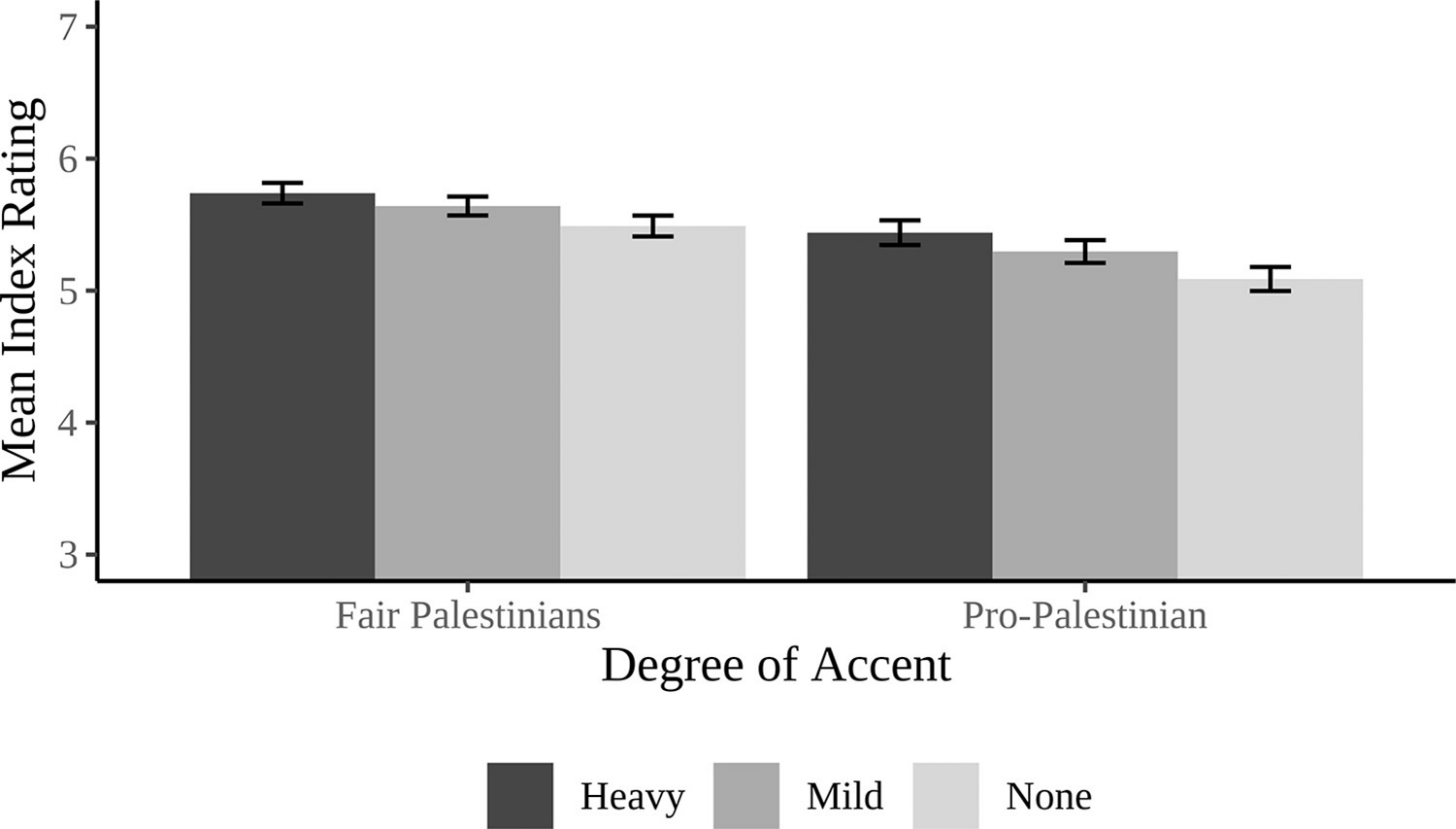
Mean ratings of the extent to which the proposal is viewed as pro-Palestinian and as fair to Palestinians as a function of the accent of the speaker presenting the proposal.

To assess the extent to which the proposal was viewed as favorable to Palestinians, two separate ANOVAs were run on the pro-Palestinian and fair to Palestinian items. However, the Cronbach’s alpha did not reach the 0.80 threshold we preregistered in order to collapse the measures into a single index (Cronbach’s α = 0.63). Therefore, each item was analyzed separately. Beginning with the extent to which the proposal was viewed as pro-Palestinian, the proposal was evaluated as being similarly pro-Palestinian across accent conditions (Native-like: *M =* 5.09, *SD* = 1.37, Mild: *M =* 5.30, *SD* = 1.29, Heavy: *M =* 5.44, *SD* = 1.40; *F*(2, 445) = 2.58, p = 0.08, η_p_^2^ = 0.01). Similarly, there was no main effect of accent on the extent to which the proposal was viewed as fair for Palestinians, (*F*(2, 445) = 1.85, *p* = 0.16, η_p_^2^ = 0.01), with participants giving similar ratings of how fair the proposal is for Palestinians across accent conditions (Heavy: *M* = 5.74, *SD* = 1.17, Mild: *M =* 5.64, *SD =* 1.07, Native-like: *M =* 5.49, *SD =* 1.18; see Fig 3).

Lastly, participants reported the extent to which they thought the proposal was a good basis for negotiations between the two sides. There was a significant effect of accent (*F*(2, 445) = 3.06, *p* = 0.05, η_p_^2^ = 0.01), although further post-hoc contrasts revealed this significant effect was driven by a marginal, but non-significant, difference in evaluating the proposal as being a good basis for negotiations between the Heavy and Native-like accent conditions (Heavy: *M* = 3.48, *SD* = 1.91; Native-like: *M* = 3.95, *SD* = 1.91; *t* = 2.18, *p* = 0.08, *d* = 0.20). For the Mild accent condition (*M* = 3.84, *SD* = 1.90), there were no significant differences detected between the Mild and Native-like (*t* = 1.10, *p* = 0.52, *d* = 0.05) or Mild and Heavy (*t* = 1.05, p = 0.55, *d* = 0.16) conditions.

In sum, accent influenced how the trust-building measures were evaluated by the Jewish-Israeli audience. Specifically, when the Palestinian delegate spoke with heavy accent, the proposed measures were judged as being significantly worse for Israelis than when they were presented by a Palestinian delegate with no detectable accent. To better understand what is driving this accent effect, we then examined how the participants evaluated the speaker, how they emotionally responded to the proposal, and the perceived fluency of the proposal depending on the accent of the Palestinian delegate.

Because there were no significant differences in proposal evaluations detected between the Mild accent condition and the other two accent conditions, the subsequent analyses will only report differences between the Native-like and Heavy accent conditions for brevity. See S2 Table for further details of the full analyses including comparisons to the Mild accent condition.

### Delegate evaluations

To understand how participants evaluated the delegate we grouped their trait evaluations into four categories: Competence (intelligent, educated, successful, and competent), Trustworthiness (trustworthy, credible, honest, and sincere), Warmth (friendly, warm, and good-natured), and Coerciveness (manipulative, self-interested, and demanding). For three of these measures–Competence (Cronbach’s α = 0.86), Trustworthiness (Cronbach’s α = 0.94), and Warmth (Cronbach’s α = 0.86)–Cronbach’s alpha met the preregistered requirement of at least 0.80 to warrant collapsing them into a single index item. However, for the Coerciveness items–namely the extent to which the Palestinian delegate was viewed as manipulative, self-interested, and demanding–only achieved a Cronbach’s alpha of 0.73 and hence each of these items was analyzed separately.

Beginning with the competence of the delegate, there was a significant effect of Accent on the delegate’s evaluations (*F*(2, 445) = 6.07, p < 0.01, η_p_^2^ = 0.03), with participants rating the Palestinian delegate more highly in traits such as intelligence, competence, and education when he communicated in Hebrew with no detectable accent (*M* = 4.35, *SD* = 1.40) than with a heavy accent (*M* = 3.82, *SD* = 1.30; *t =* 3.39, *p* < 0.01, *d* = 0.32). Participants also rated the delegate as warmer depending on Accent (*F*(2, 445) = 3.52, *p* = 0.03, η_p_^2^ = 0.01), with participants rating the Palestinian delegate more highly in traits such as friendliness, warmth, and being good-natured when they communicated in Hebrew with no detectable Arabic accent (*M* = 3.94, *SD* = 1.32) than in heavily Arabic accented Hebrew (*M* = 3.54, *SD* = 1.41; *t* = 2.42, *p* = 0.04, *d* = 0.24). However, there were no significant differences in how participants rated the trustworthiness of the delegate (*F(*2, 445) = 2.07, p = 0.13, η_p_^2^ = 0.01), with the Palestinian delegate receiving similar ratings in traits such as trustworthy, honest, and sincere across accent conditions (Native-like: *M* = 3.45, *SD =* 1.53, Heavy: *M =* 3.13, *SD* = 1.49).

Similarly, there were no significant differences in the extent to which participants viewed the Palestinian delegate as manipulative, demanding, or self-interested across accent conditions. Specifically, while there was a significant main effect of Accent in how manipulative the Palestinian delegate was viewed (*F*(2, 445) = 3.83, *p* = 0.02, η_p_^2^ = 0.01), this effect was driven by a marginally significant difference in manipulativeness ratings between the Mild (*M* = 3.69, *SD* = 1.74) and Heavy (*M* = 4.24, *SD* = 1.80; *t* = 2.19, *p* = 0.07, *d* = 0.25) accent conditions with there being no significant differences in manipulativeness ratings between the Native-like *(M* = 3.96, *SD* = 1.94) and Mild (*t* < 1) and Heavy accent conditions (*t* = 1.30, *p* = 0.40, *d* = 0.13). Furthermore, there were no significant differences across the accent conditions in the extent to which participants viewed the Palestinian delegate as looking out for his own interests (Native-like: *M* = 5.37, *SD* = 1.55, Heavy: *M* = 5.68, *SD =* 1.38; *F*(2, 445) = 1.95, *p* = 0.14, η_p_^2^ = 0.01) nor in how demanding the delegate was viewed as being (Native-like: *M* = 3.79, *SD* = 1.83, Heavy: *M* = 3.95, *SD =* 1.72; *F*(2, 445) = 2.57, *p* = 0.08, η_p_^2^ = 0.01).

### Delegate prototypicality

To measure how prototypical of a group member the Palestinian delegate was viewed, participants answered a question in which they selected which of six diagrams best represented how closely the Palestinian delegate was related to Palestinian people more broadly. Participants rated the Palestinian delegate as significantly more prototypical to other Palestinians depending on his accent (*F*(2, 445) = 7.51, *p* < 0.001, η_p_^2^ = 0.03), rating him as less prototypical when he had no detectable Arabic accent (*M* = 3.79, *SD* = 1.73) compared to when he had a heavy accent (*M* = 4.45, *SD =* 1.63; *t* = 3.53, *p* < 0.01, *d* = 0.33).

### Processing disfluency

To capture the perceived disfluency of the Palestinian delegate, participants rated how easy and clear the Palestinian delegate was when delivering the proposal (Cronbach’s α = 0.95). There was a significant effect of accent on processing disfluency (*F* (2, 445) = 6.83, *p* = 0.001, η_p_^2^ = 0.03), with participants rating the heavily Arabic accented speaker (*M* = 5.25, *SD* = 1.61) as less fluent than the speaker with no detectable Arabic accent (*M* = 5.68, *SD* = 1.33; *t* = 2.41, *p* = 0.04, *d* = 0.23).

### Emotional response

To examine the influence of accent on how participants felt in response to the proposal, two index measures were generated, one positive emotions (sympathy, empathy, hope, and optimism; Cronbach’s α = 0.93) and the other for negative emotions (anger, hatred, hostility, fear, concern, a sense of threat, disgust, and contempt; Cronbach’s α = 0.91). Participants reported that the proposal evoked similar degrees of positive (Native-like: *M* = 3.19, *SD* = 1.60, Heavy: *M* = 2.85, *SD =* 1.55; F(2, 445) = 2.53, *p* = 0.08, η_p_^2^ = 0.01) and negative (Native-like: *M* = 2.70, *SD* = 1.49, Heavy: *M* = 2.96, *SD =* 1.43; F(2, 445) = 2.05, *p* = 0.13, η_p_^2^ = 0.01) emotions across the different accent conditions.

### Evaluation of the theoretical accounts

Our results are inconsistent with the *language incongruency account*, as this account predicts that proposal evaluations would be harsher when the delegate has a native-like accent, which is the opposite of what we found. The results are consistent with the *accent bias account*. This theory predicted that the proposal would be evaluated as worse for Israelis when delivered by a heavily-accented delegate than one with no detectable accent in Hebrew. Key to this account was that because individuals judge the Palestinian delegate more harshly when he had a heavy accent, as a result they evaluate the proposal itself more negatively.

To evaluate the mechanism of the *accent bias account*, we conducted a series of mediation analyses. We assessed whether more negative proposal evaluations indeed resulted from harsher evaluations of the heavier Arabic-accented Hebrew delegate himself. Consistent with this account, participants did judge the Palestinian delegate more harshly in both competence and warmth when he spoke in a heavily-accented Hebrew as compared to when he spoke Hebrew with no detectable accent. Therefore, we conducted a multi-mediation analysis using the bootstrapping method with 10,000 simulations to assess the separate indirect effects of perceived competence and warmth of the Palestinian delegate on ratings of the proposal as more favorable for Israelis when offered in Hebrew with no non-native accent as compared to a heavy accent. Again, political attitudes were included as a covariate.

**Fig 4:**
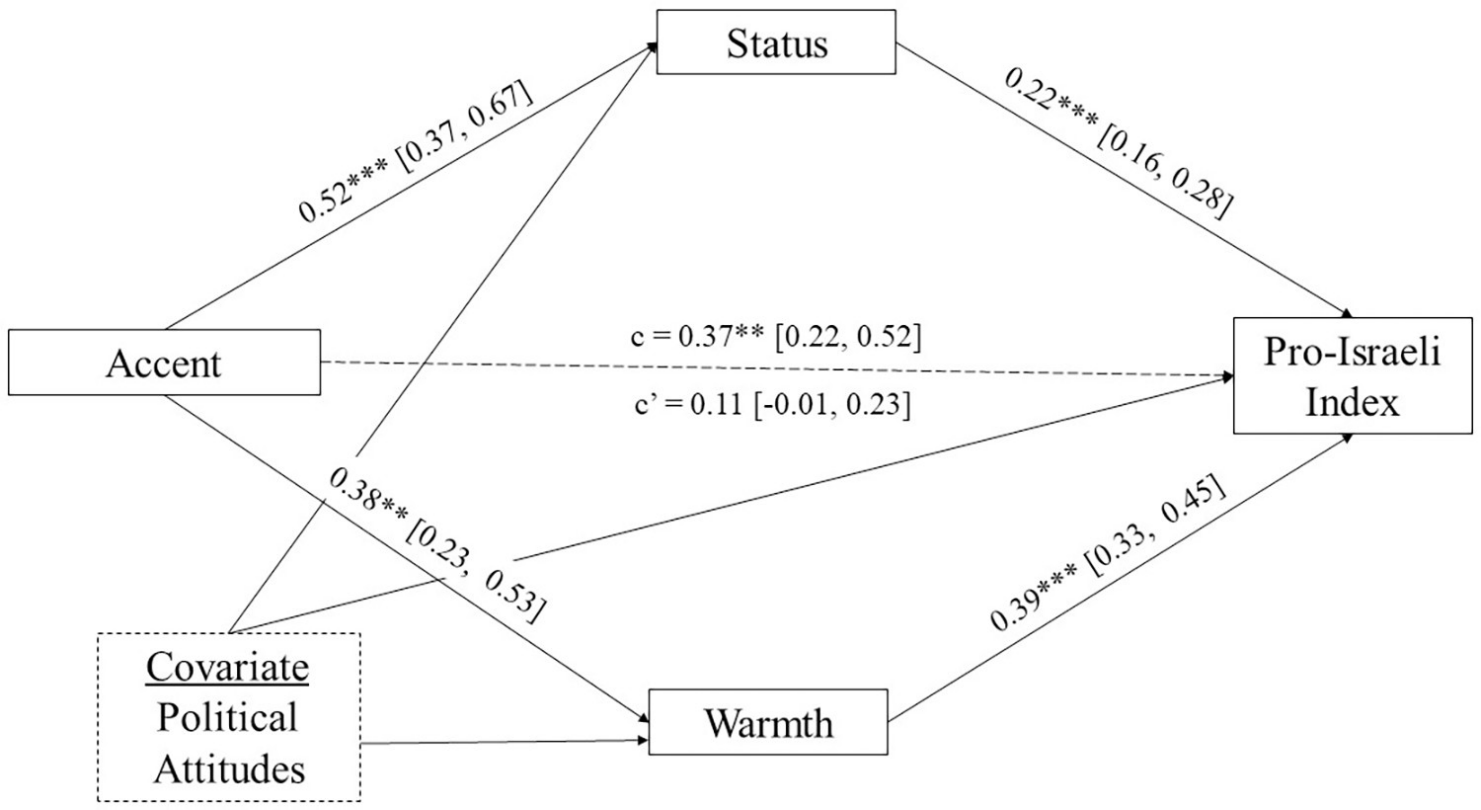
Mediation analysis of the indirect effects of perceived competence and warmth of the Palestinian representative on the direct effect of accent on pro-Israeli index scores (with political attitudes as a covariate). Mediation coefficients above refer to unstandardized coefficients. *Note*. * p< 0.05 ** p < 0.01, *** p < 0.001.

The effect of accent on the evaluation of the Palestinian proposal favorability was rendered non-significant when controlling for perceived competence and warmth of the Palestinian delegate (from *b* = 0.37, 95% CI [0.22, 0.52] to *b* = 0.11, 95% CI [S-0.01, 0.23]), consistent with a full mediation. Furthermore, perceived competence had an estimated indirect effect of 0.12 [0.07, 0.17], while perceived warmth had an estimated indirect effect of 0.15 [0.08, 0.22]. These findings suggest that when participants heard the proposal in heavily Arabic-accented Hebrew, they viewed the Palestinian representative as both lower in competence (e.g. as less intelligent, educated, and successful) and warmth (e.g. as less friendly, warm, and good-natured), which in turn decreased the perceived favorably of the proposal for Israelis (see Fig 4).

We next examined why the heavier, Arabic-accented speaker was evaluated less favorably than the speaker with no detectable Arabic accent. As we mentioned, there are several theories for why heavier, non-native accented speech typically results in less favorable evaluations of the speaker. One explanation–that negative social evaluations when encountering non-native speech are driven by processing disfluency–was not supported because accent did not affect emotional response to the proposed measures. The prototypicality explanation, which posits that individuals who communicate through heavier, non-native accented speech are evaluated as more prototypical members of their group, was consistent with the data. Participants judged the heavy, non-native accented delegate as more prototypical and they evaluated him more harshly in both competence and warmth as compared to when the delegate spoke with no detectable non-native accent. This can have negative implications for the speaker when individuals harbor negative, stereotypical attitudes about the group to which the speaker belongs, as the heavier, non-native accented speech can more saliently activate these negative attitudes.

Therefore, we used a serial mediation model to assess whether differences in the perceived prototypicality of the Palestinian delegate across accent conditions influenced evaluations of the perceived competence or warmth of the delegate, which in turn explained differences in proposal evaluations. First, consistent with the requirements of serial mediation [21], we tested whether there was a significant positive association between prototypicality and perceived competence or warmth of the speaker by regressing accent and prototypicality on each of the trait dimensions to obtain the effect of prototypicality while controlling for the effects of accent condition. Inconsistent with serial mediation, there was no significant main effect of perceived prototypicality on either perceived competence or warmth of the Palestinian delegate (*t*s < 1). Therefore, while the Palestinian delegate was judged more harshly in both competence and warmth when he spoke with a heavier, non-native accent, which in turn explained differences in how the proposal was judged, these more negative trait evaluations of the speaker were not due to differences in the perceived prototypicality of the delegate across accent conditions.

## Discussion

We found that accent can impact diplomacy: It can inadvertently influence how people respond to trust-building proposals. Specifically, when native Hebrew speakers heard a proposal from a Palestinian delegate with a heavily Arabic-accented Hebrew, they judged him more harshly in both perceived competence and warmth compared to when the Palestinian delegate spoke Hebrew with no detectable Arabic accent. This, in turn, led Israeli participants to judge the proposal as less favorable for them. These findings provide support for the *accent bias account*, according to which negative evaluations typical of more heavily accented, non-native speakers can impact how people judge the information that is communicated. Our findings also provide strong evidence against the *language incongruency account*, which predicted that the incongruency between the speaker identity and speech style when communicating in Hebrew with no detectable accent would result in more negative evaluations of the Palestinian delegate, which in turn would result in less favorable judgments of the proposal.

This study provided a partial support for an explanation of the *accent bias account*. As this account suggests, we found that the reason the proposal from the heavily accented speaker was perceived more negatively was that he himself was perceived more negatively, specifically as less competent and less warm. But the two explanations for why a more negative perception of the delegate led to more negative perception of the proposal were not supported. The first was that individuals judge heavier accented speakers more negatively due to processing disfluency, with the accent triggering a more negative affective response. We found no evidence for this. The second explanation was that the delegate was perceived more negatively because he was perceived as a more prototypical group member. While the heavier accented speaker was indeed perceived as a more prototypical member, we found no evidence that this led to a more negative perception of him as a person.

It is therefore an open question why people evaluate speakers more negatively when they communicate through heavier, non-native accented speech. One possible explanation, in line with communication accommodation theory, is that rather than signaling group prototypicality, speaking with a heavy accent serves as a form of speech divergence from the speech style of the recipients. Particularly with a heavy accent, it may be the case that listeners perceive a speaker who is communicating with such an accent as not trying hard enough to communicate clearly [8]. Alternatively, it may be that rather than the accent activating group-specific stereotypes, the delegate is being more harshly judged due to stereotypes regarding non-native speakers more generally. This would be in line with Lindemann (2003), who found that native listeners rated a non-native speaker as lower in status even though most individuals were unable to correctly identify the specific non-native accent they were listening to [22].

While our findings illustrate that accent bias can occur even when the speaker is offering a proposal that presents a possible benefit to the recipient, it might be important to investigate ways to mitigate these negative social evaluations of accent given its impact on the evaluation of the information itself. To do this, we first need to fully understand the root cause of the bias. Non-native accent is typical of individuals communicating through a language that is not native to them, and often lingers after all other facets of language production reach a high degree of fluency [23]. Therefore, harsh judgments of the speaker by native listeners are unwarranted, particularly if these negative social evaluations arise from perception that accent is due to lack of effort or from inferences about lower intelligence or character of the non-native speakers. Hence, future studies may examine ways to alleviate these negative evaluations of heavily accented non-native speakers. For example, it might be important to investigate whether the type of bias that results from accent is inevitable in a violent conflict or is malleable and conducive to interventions.

It might also be of interest to examine how Israelis respond to trust-building measures offered through non-native accents beyond Palestinian Arabic accented Hebrew. By doing so, one could evaluate if native Hebrew speaking Israeli listeners evaluate trust-building measures more negatively when offered through heavy, non-native accented Hebrew more broadly, or if this accent effect is specific to listening to heavily accented, non-native speech of an adversary. This may provide fruitful insights into how Israelis may respond to trust-building measures offered by a mediating party that also has a heavy, non-native accent in Hebrew. While the current status of the Israeli-Palestinian conflict does not allow for such investigations, we hope that future researchers will explore these questions once it is possible to resume trust-building efforts.

Finally, there are several ways in which our findings could be extended to better understand the influence of accent on receptiveness to trust-building efforts. For instance, it would be important to investigate this question with other ongoing intergroups conflicts. While the focus of this study–the Israeli-Palestinian conflict–serves as a prime example of violent, protracted conflict, parties engaged in intergroup conflict not sharing a native language is the rule rather than the exception. For example, similar to the Israeli-Palestinian conflict, the Kashmir conflict is a protracted conflict which has been ongoing for decades and involves parties who speak multiple different native languages such as Kashmiri and Dogri, Urdu, and Hindi [24]. Examining how accent influences trust-building efforts in different conflicts which vary in, amongst other factors, the languages, cultures, and power dynamics of the parties, would be essential to fully understanding the ramifications of accent in shaping attitudes regarding conflict de-escalation efforts.

## Conclusion

Understanding factors that contribute to violent intergroup conflict is essential to reducing the human suffering imposed by ongoing war, and it might allow us to find ways to bridge those divides. This study contributes to the growing literature demonstrating the importance of communication-based interventions in deescalating conflict [15, 25, 26]. Specifically, demonstrating that changing something as minor as the accent of a speaker in negotiations between the sides can significantly shift attitudes in trust-building initiatives. This research has important implications for the de-escalation and resolution of ethnopolitical conflicts. Specifically, given that individuals view trust-building measures in cross-national conflict more favorably when presented without a detectable, non-native accented speech, one could leverage this information to increase the chances of a proposal being favorably received thereby facilitating conflict resolution.

## Supporting information

S1 File Accent pretest survey methods and results. (DOCX)

S1 Table Mean accent ratings and perceived age of the speaker from norming study (study 4). Higher scores for mean accent rating indicate the speaker was rated as having heavier, Arabic-accented Hebrew.
(DOCX)

S2 Table Mean ratings, standard deviations, omnibus tests, and post-hoc Tukey contrasts for each index measure. (DOCX)
