# Supplementary material for: The influence of accent on the evaluation of trust-building efforts during conflict
Source: PLoS One. 2024 Nov 13;19(11):e0311373. doi: 10.1371/journal.pone.0311373 (PMC11560000; doi:10.1371/journal.pone.0311373)
Supplement: S1 File — (DOCX) [file pone.0311373.s001.docx]

**Supporting Information**

**S1 File. Accent pretest survey methods and results.**

Once the proposal was finalized, eleven male speakers between the ages of 35 to 55 years old recorded the proposal in a neutral tone. Of these speakers, 9 were native Palestinian Arabic speakers who spoke Hebrew as a foreign language and 2 were native Hebrew, Israeli speakers. The age range was chosen to ensure the speaker seemed like a credible, realistic source given that listeners can often detect the age of a speaker from their voice (Hughes & Rhodes, 2010). The 9 native Arabic speakers all self-identified as speaking Palestinian Arabic as their native language, which is a dialect used by Arabs and Palestinians from Syria, Lebanon, Palestine, and Israel [1]. Palestinian Arabic is characterized by gutturals – or ‘letters of the throat’ – sounds produced with a primary constriction of the posterior regions of the vocal tract [2-4]. Along with other differences between Palestinian Arabic and modern Hebrew, modern Hebrew does not have such guttural sounds, and thus native Palestinian Arabic speakers who tend to import these characteristics more extensively when speaking Hebrew tend to be perceived as having heavier Palestinian Arabic-accented Hebrew.

Additionally, while the proposal is framed as being from a Palestinian representative for all conditions, the two native Hebrew, Israeli speakers were selected for the native-like Hebrew accented condition. This practice of using a native speaker under the guise of being a non-native one is often utilized, both because the majority of non-native speakers often speak through at least mildly non-native accented speech and, if the identity of the speaker is known, listeners will often nonetheless hear a detectable, non-native accent from native speaker recordings if non-native speech is expected [5]. These initial recordings were then processed using Audacity (https://www.audacityteam.org/) and normalized to a similar perceived volume across speakers.

From these processed recordings, a brief, 10 second clip was taken from each recording to act as a sample, and a norming study was conducted with 120 native Hebrew speakers born and currently residing in Israel (*M*_Age_ = 37.41, 49.14% Women). In the norming study, following an initial audio check to ensure their audio equipment was turned on and functioning, participants were randomly assigned to rate three of the 11 speakers on the perceived degree of Arabic-accented Hebrew and age of the speaker. To do so, for each audio clip participants listened to the sample clip and then immediately following the clip rated how accented they perceived the speaker as being (on a scale from 1 (*no detectable Arabic accent*) to 7 (*very heavy Arabic accent*)) and then provided a guess of the speaker’s age.

Because one of the two native-like Hebrew speakers was rated as having a slightly heavier accent than the other (Speaker 11), a new native Hebrew speaker was recorded and processed and a second group of 40 native Hebrew speakers was recruited (M_Age_ = 37.63, 57.90% Women). In this follow up norming study, participants listened to the new native Hebrew speaker (Speaker 12) and Speaker 4 and Speaker 9 from the initial norming study in a randomized order. To ensure accent ratings were comparable across the two norming studies, accent ratings for Speaker 4 and Speaker 9 were compared and did not yield a significant difference between the initial and follow up norming study ratings for each of those speakers respectively (*t*s < 1). Therefore, data for Speaker 12 was used in the final norming dataset (see S2 Table for more details).

After reviewing the final norming data, Speakers 2 and 4 were selected the heavily Arabic-accented Hebrew speakers, Speaker 7 and 9 as the mildly Arabic-accented Hebrew speakers, and Speaker 10 and 12 as the native-like Hebrew speakers. An ANOVA comparing ratings of accent across the assigned accent conditions confirmed the speakers were rated as having significantly different levels of perceived accent in Hebrew (*F*(2, 193) = 253.90, *p* < 0.001, η_p_^2^ = 0.73), with further paired t-tests revealing significant differences between each of the accent conditions (Heavy vs. Mild: *p* < 0.001; Heavy vs. Native-like: *p* < 0.001; Mild vs. Native-like: *p* < 0.001). Lastly, a final set of paired t-tests yielded similar perceived accent ratings of the speakers within each accent condition (*t*s < 1).

**References**

1. Bin-Muqbil, M. Phonetic and phonological aspects of Arabic emphatics and gutturals (Publication No. 3222872). Ph.D. Dissertation, The University of Wisconsin-Madison. 2006. Available from: http://proxy.ulib.csuohio.edu/login?url=https://www.proquest.com/dissertations-theses/phonetic-phonological-aspects-arabic-emphatics/docview/304975410/se-2
2. Jarrar, M, Habash, N, Alrimawi, F, Akra, D, & Zalmout, N. Curras: an annotated corpus for the Palestinian Arabic dialect. Language Resources and Evaluation*.* 2017;51: 745-775. doi: 10.1007/s10579-016-9370-7
3. Mashaqba, B, Huneety, A, & Abu Guba, MN, & Al-Duneibat, B. Production of gutturals by non-native speakers of Arabic. Indonesian Journal of Applied Linguistics. 2022;12(2): 334-347. doi: 10.17509/ijal.v12i2.26143
4. Watson, JCE. The phonology and morphology of Arabic. Oxford, UK: Oxford University Press; 2002. doi: 10.1093/oso/9780199257591.001.0001
5. Rubin, DL. Nonlanguage factors affecting undergraduates' judgments of nonnative English-speaking teaching assistants. Research in Higher Education, 1992;33: 511-531. doi: 10.1007/BF00973770
