# Supplementary material for: The influence of accent on the evaluation of trust-building efforts during conflict
Source: PLoS One. 2024 Nov 13;19(11):e0311373. doi: 10.1371/journal.pone.0311373 (PMC11560000; doi:10.1371/journal.pone.0311373)
Supplement: S1 Table — Higher scores for mean accent rating indicate the speaker was rated as having heavier, Arabic-accented Hebrew. (DOCX) [file pone.0311373.s002.docx]

**S1 Table. Mean accent ratings and perceived age of the speaker from norming study (Study 4). Higher scores for mean accent rating indicate the speaker was rated as having heavier, Arabic-accented Hebrew.**

| **Speaker** | **Number of Ratings** | **Mean Accent Rating** | **Mean Perceived Age** |
| --- | --- | --- | --- |
| 1 | 34 | 5.00 | 42.94 |
| 2 | 31 | 5.68 | 41.58 |
| 3 | 32 | 4.63 | 39.44 |
| 4 | 31 | 5.55 | 34.45 |
| 5 | 34 | 3.62 | 35.06 |
| 6 | 31 | 4.74 | 36.62 |
| 7 | 26 | 4.04 | 35.08 |
| 8 | 37 | 5.47 | 25.70 |
| 9 | 28 | 3.61 | 32.04 |
| 10 | 36 | 1.42 | 32.75 |
| 11 | 28 | 2.43 | 42.18 |
| 12 | 38 | 1.34 | 34.76 |
