# Supplementary material for: The influence of accent on the evaluation of trust-building efforts during conflict
Source: PLoS One. 2024 Nov 13;19(11):e0311373. doi: 10.1371/journal.pone.0311373 (PMC11560000; doi:10.1371/journal.pone.0311373)
Supplement: S2 Table — (DOCX) [file pone.0311373.s003.docx]

**S2 Table. Mean ratings, standard deviations, omnibus tests, and post-hoc Tukey contrasts for each index measure.**

| **Index** | **Descriptive Statistics** | ***F*-test** | **Native-like vs. Mild** | **Mild vs. Heavy** |
| --- | --- | --- | --- | --- |
| **Status** | Native-like: 4.35(1.40)  Mild: 4.10(1.39)  Heavy: 3.82(1.30) | *F*(2, 445) = 6.07,  *p* < 0.01, η_p_^2^=0.03 | *t* = 1.97,  *p* = 0.12,  *d* = 0.15 | *t* = 1.38,  *p* = 0.36,  *d* = 0.17 |
| **Solidarity** | Native-like: 3.45(1.53)  Mild: 3.32(1.44)  Heavy: 3.13(1.49) | *F(*2, 445) = 2.07,  *p* = 0.13, η_p_^2^=0.01 |  |  |
| **Warmth** | Native-like: 3.94(1.32)  Mild: 3.76(1.43)  Heavy: 3.54(1.41) | *F*(2, 445) = 3.52,  *p* = 0.03, η_p_^2^=0.01 | *t* = 1.10,  *p* = 0.52,  *d* = 0.10 | *t* = 1.05,  *p* = 0.55,  *d* = 0.13 |
| **Manipulative** | Native-like: 3.96(1.94)  Mild: 3.69(1.74)  Heavy: 4.24(1.80) | *F*(2, 445) = 3.83,  *p* = 0.02, η_p_^2^=0.01 | *t*  < 1 | *t* = 2.19,  *p* = 0.07,  *d* = 0.25 |
| **Self-Interested** | Native-like: 5.37(1.55)  Mild: 5.43(1.46)  Heavy: 5.68(1.38) | F(2, 445) = 1.95,  *p* = 0.14, η_p_^2^=0.01 |  |  |
| **Demanding** | Native-like: 3.79(1.83)  Mild: 3.50(1.68)  Heavy: 3.95(1.72) | F(2, 445) = 2.57,  *p* = 0.08, η_p_^2^=0.01 |  |  |
| **Source Prototypicality** | Native-like: 3.79(1.73)  Mild: 4.40(1.56)  Heavy: 4.45(1.63) | *F*(2, 445) = 7.51,  *p* < 0.001, η_p_^2^=0.03 | *t* = 3.13,  *p <* 0.01,  *d* = 0.31 | *t <* 1 |
| **Processing Disfluency** | Native-like: 5.68(1.33)  Mild: 5.03(1.63)  Heavy: 5.25(1.61) | *F* (2, 445) = 6.83,  *p* = 0.001, η_p_^2^=0.03 | *t* = 3.67,  *p <* 0.001,  *d* = 0.35 | *t* = 1.37,  *p* = 0.36,  *d* = 0.11 |
| **Positive Emotions** | Native-like: 3.19(1.60)  Mild: 3.17(1.66)  Heavy: 2.85(1.55) | F(2, 445) = 2.53,  *p* = 0.08, η_p_^2^=0.01 |  |  |
| **Negative Emotions** | Native-like: 2.70(1.49)  Mild: 2.69(1.45)  Heavy: 2.96(1.43) | F(2, 445) = 2.05,  *p* = 0.13, η_p_^2^=0.01 |  |  |

*Note.* Post-hoc Tukey contrasts only provided when the *F*-test is significant (*p* < 0.05)
